# Effect of a transcultural nursing course on improving the cultural competency of nursing graduate students in Korea: a before-and-after study

**DOI:** 10.3352/jeehp.2023.20.35

**Published:** 2023-12-04

**Authors:** Kyung Eui Bae, Geum Hee Jeong

**Affiliations:** 1 Division of Nursing Science, Dongseo University, Busan, Korea; 2 School of Nursing and Research Institute in Nursing Science, Hallym University, Chuncheon, Korea; Hallym University, Korea

**Keywords:** Cultural competency, Curriculum, Graduate nursing education, Nursing students, Republic of Korea

## Abstract

**Purpose:**

This study aimed to evaluate the impact of a transcultural nursing course on enhancing the cultural competency of graduate nursing students in Korea. We hypothesized that participants’ cultural competency would significantly improve in areas such as communication, biocultural ecology and family, dietary habits, death rituals, spirituality, equity, and empowerment and intermediation after completing the course. Furthermore, we assessed the participants’ overall satisfaction with the course.

**Methods:**

A before-and-after study was conducted with graduate nursing students at Hallym University, Chuncheon, Korea, from March to June 2023. A transcultural nursing course was developed based on Giger & Haddad’s transcultural nursing model and Purnell’s theoretical model of cultural competence. Data was collected using a cultural competence scale for registered nurses developed by Kim and his colleagues. A total of 18 students participated, and the paired t-test was employed to compare pre-and post-intervention scores.

**Results:**

The study revealed significant improvements in all 7 categories of cultural nursing competence (P<0.01). Specifically, the mean differences in scores (pre–post) ranged from 0.74 to 1.09 across the categories. Additionally, participants expressed high satisfaction with the course, with an average score of 4.72 out of a maximum of 5.0.

**Conclusion:**

The transcultural nursing course effectively enhanced the cultural competency of graduate nursing students. Such courses are imperative to ensure quality care for the increasing multicultural population in Korea.

## Graphical abstract

[Fig f2-jeehp-20-35]

## Introduction

### Background/rationale

The number of families with multicultural backgrounds in Korea continues to rise annually. According to Statistics Korea, the total population of Korea peaked at 51,829,136 in 2020, compared to 51,629,512 in 2018, and subsequently fell to 51,628,117 in 2022. This indicates a decline of 201,019 in 2 years and a total decline of 1,395 in the last 5 years. Conversely, the number of people from multicultural backgrounds increased from 1,008,520 in 2018 to 1,151,004 in 2022. This reveals a growth of 155,466 individuals (14.1%) within the last 5 years [1]. As the number of multicultural individuals seeking medical treatment is on the rise, it is crucial for Korean nurses to understand the cultural heritage of these patients and their caregivers to deliver top-notch nursing care. When dealing with multicultural populations in nursing practice, there may be communication challenges arising from language differences. Additionally, problems may arise when both multicultural population and healthcare providers fail to understand cultural differences [2]. Nurses and healthcare providers need cultural nursing competence to understand and respect individuals with varied cultural backgrounds and lifestyles so that they can deliver high-quality patient-centered nursing services [3]. A cultural nursing course was offered to undergraduate students in Korea, resulting in a significant increase in their overall cultural competence scores, as well as in each category, including cultural awareness, cultural knowledge, cultural sensitivity, and cultural skills (P<0.001) after the course [4]. However, there has been no evaluation of this course for graduate nursing students in Korea.

### Objectives

This study aimed to investigate the effect of a transcultural nursing course on enhancing the cultural competency of students in a graduate nursing program in Korea. The specific objectives were as follows: first, to evaluate changes in cultural competency among participants following the completion of a transcultural nursing course in various areas, including communication, biocultural ecology and family, dietary habits, death rituals, spirituality, equity, and empowerment and intermediation; and second, to determine the overall satisfaction of the participants upon completion of the course.

## Methods

### Ethics statement

The researcher (G.H.J.) clarified the investigation’s objectives, and the participants were assured of their anonymity and data confidentiality. It was emphasized that the data would be used solely for research purposes, and they were informed of their right to opt-out at any point during the survey. The students who agreed to participate provided their informed consent.

### Study design

This was a before-and-after study. The main text was described according to the Transparent Reporting of Evaluations with Nonrandomized Design (TREND) statement available from: https://www.cdc.gov/trendstatement/index.html.

### Setting

This study, including an educational intervention was conducted as part of the graduate program at the School of Nursing, Hallym University, in Chuncheon, Korea from March to June 2023. Participants were administered a survey questionnaire prior to the educational intervention, and the same questionnaire was distributed after the intervention for all participants, with response data collected (Supplements 1, 2). The survey questionnaires were distributed through the online survey platform of Hallym University.

### Participants

A total of 20 graduate students enrolled in the first semester of a master’s program—specifically, taking the transcultural nursing course—agreed to participate in this study. They were all included in this before-and-after study. After excluding a student who consented to participate but did not complete the pre-survey and another who did not complete the post-survey, data from 18 students were analyzed.

### Interventions: development and implementation of a transcultural nursing course

The transcultural nursing course for graduate students was developed based on the transcultural nursing model of Giger and Haddad [5] (Supplement 3). Based on the model of Giger and Haddad [5], this course dealt with 6 cultural phenomena that have a profound impact on nursing care as key themes to promote the cultural knowledge and skills of graduate students. These 6 cultural phenomena were communication, space, social organization, time, environmental control, and biological variations. The model of Purnell [6] presents 4 levels of cultural competence in nursing. The lowest level of cultural competence is “unconsciously incompetent,” when one is unaware of one’s lack of knowledge about other cultures, and “consciously incompetent,” when one is aware of one’s lack of knowledge about other cultures. The next stage is “consciously competent,” which involves learning about the client’s culture and providing culturally specific interventions, and finally there is “unconsciously competent,” when one automatically cares for clients with diverse cultures. Based on the model of Purnell [6], the goal of this course was to enable graduate nursing students to assess, plan, intervene and evaluate clients from different cultures in a culturally competent manner in the clinical setting.

The teaching methods of this course included flipped learning, problem-based learning methods, online lectures, small group activities, discussions, role-plays, individual reports and presentations, and reflective activities. In the online lectures, students learned about 6 cultural phenomena and promoted cultural knowledge and self-assessment of cultural competencies through individual reports and presentations on various cases related to cultural issues. In the face-to-face lectures, which used flipped learning, the online learning was completed before the lecture, and small group discussions and presentations were held. Students presented their cases of caring for patients from different cultures in the actual nursing setting, sharing their difficulties and effective nursing strategies and plans. In the case of a Vietnamese immigrant to Korea and a Korean elderly woman who moved to Australia, they tried to find different cultural interventions by using role-playing and problem-based learning methods together.

The curriculum’s structure and content were evaluated by a team of 5 faculty experts who have previously taught transcultural nursing courses at various universities. This course was compulsory and was required during the first semester of the 2023 academic year at Hallym University’s Graduate School of Nursing Education. It consisted of 15 two-hour sessions, comprising 8 hours of non-face-to-face online lectures; 20 hours of face-to-face small group discussions and presentations; and 2 hours for an exam and curriculum evaluation by students (Supplement 2).

### Outcomes

The outcome variables were 7 subscales of the questionnaire, including communication, biocultural ecology and family, dietary habits, death rituals, spirituality, equality, and empowerment and intermediation.

### Data sources/measurement

The data source was the raw response data from 18 participants (Dataset 1). The survey comprised 8 demographic characteristics, 35 items on cultural competence measurement, and 3 items on satisfaction and feedback after the course.

The demographic characteristics consisted of age, gender, type of affiliated hospital, current position, career experience, experience of cultural nursing care, the experience of receiving cultural nursing care education, and overseas experience.

Cultural competence was measured using the Cultural Competence Scale for Registered Nurses (CCS-RN) developed by Kim et al. [7], which is available under the Creative Commons license (Supplement 1). The CCS-RN tool consists of 35 items with 7 subscales, including communication (6 items), biocultural ecology and family (9 items), dietary habits (3 items), death rituals (3 items), spirituality (3 items), equity (5 items), and empowerment and intermediation (6 items). Each item is answered on a 5-point Likert scale ranging from 1 for “not at all” to 5 for “very much so.” A higher mean score indicates greater cultural competence. The item-level content validity index of this measurement tool by 5 nurses with experience in cultural nursing care ranged from 0.8 to 1.0 for all items. Its internal reliability, expressed as a Cronbach’s ⍺ value (95% confidence interval [CI]) was 0.94 (95% CI, 0.93–0.95), with a minimum of 0.77 (95% CI, 0.73–0.81) and a maximum of 0.90 (95% CI, 0.88–0.91) for the subscales in the study by Kim et al. [7]. Cronbach’s ⍺ before the intervention in the present study was 0.98 (P<0.05), and it was 0.97 (P<0.05) after the intervention.

Participants’ overall satisfaction with the course was measured on a 5-point Likert scale, ranging from 1 for “very dissatisfied” to 5 “very satisfied.” Participants’ positive feedback and suggestions for improvements to the course were measured with one open-ended question each.

### Bias

There was no selection bias because all target participants agreed to participate in the survey.

### Study size

Because all target students were included, prior sample size estimation was not done. Post-hoc power analysis for differences between 2 dependent means (matched pairs) showed a power (1-β err probability) 0.9953 using G*Power (http://www.gpower.hhu.de/) based on the following parameters: 2-tailed test; effect size of 1.1427; alpha error probability of 0.05; and a total sample size of 18. The effect size was determined from the mean and standard deviation of the before group (3.17±1.11) and the after group (4.06±0.74). The correlation between groups was 0.7141.

### Assignment method

A single-group before-and-after test was done, so that there was no specific assignment method.

### Blinding (masking)

No blinding was done since there was no control group. Although an author (G.H.J.) participated in the educational intervention, she was not involved in graduate students’ evaluation process, and the survey was done through the online system of her university. The authors made and analyzed a coding of the response results.

### Unit of analysis

All participants’ data were analyzed individually.

### Statistical methods

The comparison analysis was done for each outcome variable (category). Subscale value was a sum of corresponding items. A comparison of each category was done between subscale values before and after the intervention. The paired t-test was performed and Cronbach’s ⍺ was calculated using DBSTAT ver. 5.0 (DBSTAT Co.) available at https://www.dbstat.com.

## Results

### Characteristics of the participants

The participating graduate students were mostly women (88.9%), worked in hospitals or clinics (100.0%), and had multicultural patient care experience (83.3%). Very few had previously received cultural nursing education (5.6%) (Table 1).

### Changes in cultural nursing competence after the educational intervention

**Fig. 1.**
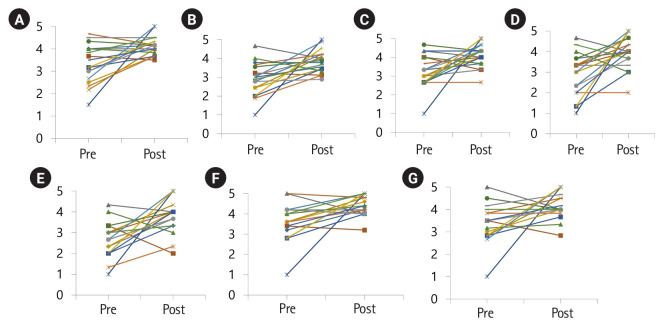
Pre- and post-test comparison of graduate nursing students’ cultural competence for 7 subscales. (A) Communication, (B) biocultural ecology and family, (C) dietary habits, (D) death rituals, (E) spirituality, (F) equity, and (G) empowerment and intermediation.

There were significant differences in the cultural nursing competence of graduate students in the total mean score and all 7 subcategories (P<0.01) (Table 2, Fig. 1, Supplement 4).

### Participants’ satisfaction, positive feedback, and comments on the course

The average score for satisfaction was 4.72 out of a maximum of 5.0. Participants’ positive feedback and comments on improvements to the course are shown in Table 3 (Supplement 5).

## Discussion

### Key results

This study aimed to evaluate the impact of a cultural nursing program on improving the cultural competency of graduate nursing students in Korea through the cultural competence scale for registered nurses. There was an improvement in the total mean score and all 7 subcategories of cultural competency. Furthermore, participants were highly satisfied with the course and gave extensive positive feedback.

### Interpretation

#### Communication

In this study, students’ communication competencies improved after participating in the course. While direct foreign language skills are important, there was a significant improvement in overall communication through increased competence in non-verbal communication and utilization of information and resource mobilization to provide information to patients.

#### Biocultural ecology and family

Cultural knowledge of the patient’s family relationships and roles is also essential for nursing care; furthermore, nurses’ understanding of biological differences and differences in disease development is critical in differentiating between normal and abnormal drug reactions. The improvement in this area is a promising result for the transcultural nursing course.

#### Dietary habits

Dietary habits relate to dietary differences according to culture, dietary provisions, and the meaning and taboos of food. When nurses care for patients with different cultures, recognizing and applying the importance of providing a culturally appropriate diet is important for the recovery of the patients’ health. Hospitalized patients experience difficulties with hospital meals; therefore, nurses’ ability to find different solutions helps improve patients’ nutrition. In this study, students’ cultural nursing competencies were enhanced to improve patients’ nutrition.

#### Death rituals

Respecting end-of-life practices and rituals is a sensitive and crucial aspect of nursing. Nurses experience difficulties in caring for the general dying population in Korea. It is essential to consider cultural differences in end-of-life-related attitudes to apply them in practice rather than just recognizing cultural differences. Students who participated in this course significantly improved their cultural competence in death rituals.

#### Spirituality

There was also a significant increase in “spirituality” related to religion. Being attuned to patients’ spiritual beliefs and practices can greatly influence care decisions and patient satisfaction.

#### Equality

The highest nursing competency score post-intervention was in the “equality” domain, where there was a significant increase in the perception of respecting the patient and providing equal care—that is, providing tailored interventions without bias and discrimination.

#### Empowerment and intermediation

This ability involves developing cultural nursing capabilities through experience in one’s knowledge, skills, and approaches to multicultural nursing. In addition, linking multicultural policies with welfare benefits and support systems is essential. The improvements in this category also showed evidence of the effectiveness of this course.

### Comparison with previous relevant studies

No other study has used the same measurement tool as that in this study for graduate students. The original scale was developed with registered nurses [7]. Additionally, the curriculum of transcultural nursing in other universities is not the same as that of this study. Therefore, it is impossible to directly compare the results of the present study to any others. However, there have been multiple studies evaluating cultural competency courses. In Japan, international nursing courses increased nursing students’ intercultural sensitivity [8]. At the Medical University in Tokyo, nursing students participated in the international nursing course. They listened to a lecture in English. They discussed, collaborated, and completed common tasks with students in the United States through online classes. That collaborative work will loosen the tension during the care for foreigners. In Korea, a standardized patient was used for improving cultural competency among undergraduate nursing students. The standardized patient and his mother were set to be from the United Arab Emirates in the simulation. Through this simulation program, nursing students’ cultural competency increased compared to that of the control group [9]. In Vietnam, when nursing students completed a cultural competence course with a field visit, they reported “obtaining cultural experiences” and “expanding understanding of cultural competence through field experience” compared to students without a field visit [10]. In various health science fields, including pharmacy, occupational therapy, health science, podiatry, and physical therapy, interventions focusing on cultural competency have proven to improve knowledge, skill execution, attitudes, and student satisfaction [11]. A study on the effectiveness of virtual simulations and problem-based learning in 61 undergraduate or postgraduate nursing students from 5 Asian countries found that students’ cultural competence increased [12]. In Iran, the cultural competence and self-efficacy of postgraduate nursing students improved after an online cultural care training program.

The training program was executed online due to coronavirus disease 2019 pandemic [13]. Four 2-hour sessions of the curriculum consisted of lectures with PowerPoint presentations, questions and answers, case scenarios, case reports, group discussions, and videos. There are other studies on the effect of transcultural nursing curriculum on nursing competency; however, the content of the curricula and measurement tools varied among studies. Although it is difficult to directly compare those studies, the curriculum used in each study should be considered for adoption at other institutions.

### Limitations

This study had a single-group pre-post-test design without a control group, which limits the interpretation of the effects.

### Generalizability

The participating graduate students in this study did not receive transcultural nursing education in their undergraduate courses. Therefore, the present results may reflect the cultural nursing competence of nurses of the same ages in Korea, who did not experience transcultural nursing education. However, it is challenging to generalize to other universities since this was a single-institution study.

### Suggestions

It is challenging to anticipate better competence in biocultural ecology and family through the present course because the topic is broad and difficult to acquire in a short period. Furthermore, competence in death rituals is also difficult, even as it relates to caring for Korean patients and their families. A more specific multicultural nursing course should be introduced to overcome the present limitations. After adopting those specific courses, new experimental studies including a control group will be able to show the benefit of those courses. Furthermore, the long-term effects of this course should be followed-up.

### Implication

Compared with undergraduate nursing students, graduate students who care for multicultural clients have had little experience with culture-related courses or education since graduating from college. There has been no curriculum evaluation on this topic in nursing graduate schools in Korea. Therefore, it is essential to develop and apply mandatory or optional courses to improve cultural competence in graduate curricula of nursing schools. As cultural diversity is increasing in Korea, cultural competency education and training should be given more attention and strengthened through university and graduate education, as well as through nursing societies, professional certification, and continuing education programs to care for patients from diverse cultural backgrounds.

### Conclusion

This course proved effective in improving the cultural competence of nursing graduate students in Korea. It is necessary to improve cultural nursing competence through the improvement of curricular content and the development of other related courses. After completing the transcultural nursing course, graduate students progressed from “unconsciously incompetent” or “consciously incompetent” before attending the transcultural nursing course to a higher level of “consciously competent” after completing the course, according to the 4 levels of cultural competence in Purnell’s model. While this course was designed for graduate students in the Master of Science in Nursing Education program, cultural competency is also important for other graduate nursing programs, such as nurse practitioner programs. Those courses will be able to help nurses care for the multicultural population in Korea. The results of this research will help develop an evidence-based curriculum and pedagogy for transcultural nursing education in Korea and other countries.

## Figures and Tables

**Figure f2-jeehp-20-35:**
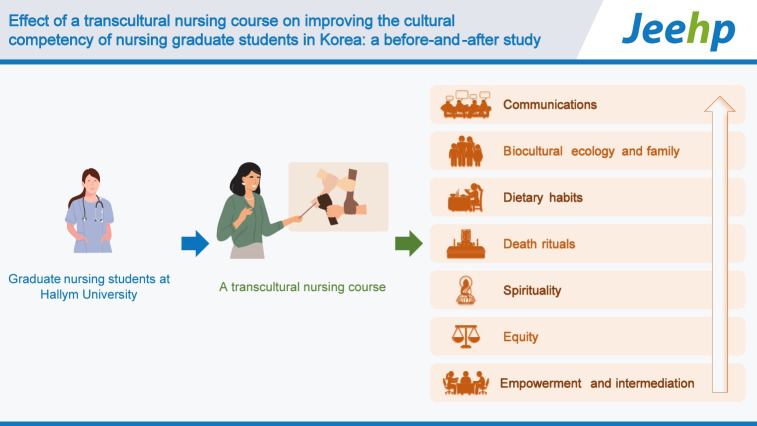


**Table 1.** Participating graduate students’ general characteristics (N=18)

Characteristic	No. (%)
Age (yr)	
20–29	2 (11.1)
30–39	6 (33.3)
≥40	10 (55.6)
Gender	
Women	16 (88.9)
Men	2 (11.1)
Type of hospital	
General hospital	17 (94.4)
Clinic	1 (5.6)
Current position	
Staff nurse	10 (55.6)
Charge & head nurse	8 (44.4)
Career (yr)	
≤5	1 (5.6)
>5 ≤10	3 (16.7)
>10 ≤20	8 (44.4)
>20	6 (33.3)
Experience of multicultural patient care	
No	3 (16.7)
Yes	15 (83.3)
Experience of receiving cultural education	
No	17 (94.4)
Yes	1 (5.6)
Experience of overseas residence	
No	18 (100.0)
Yes	0

**Table 2.** Differences in the cultural competence scale of participants before and after a transcultural nursing course in Hallym University (N=18)

Subscale	Mean±SD		Mean difference (Pre–Post)	P-value
	Pre-test	Post-test		
Communication	3.33±0.87	4.15±0.42	0.82	0.004
Biocultural ecology and family	2.90±0.87	3.85±0.60	0.95	0.003
Dietary habits	3.24±0.86	4.09±0.60	0.85	0.007
Death rituals	2.89±1.06	3.98±0.80	1.09	0.004
Spirituality	2.67±0.84	3.76±0.82	1.09	0.002
Equity	3.61±0.93	4.43±0.51	0.82	0.008
Empowerment and intermediation	3.40±0.86	4.14±0.57	0.74	0.018
Total	3.17±1.11	4.06±0.74	0.89	<0.001

SD, standard deviation.

**Table 3.** Participants’ comments after completing the course (N=18)

Category	Specific content (frequency)
Positive feedback	New opportunities for learning in diverse cultures and cultural nursing (7)
	- Opportunities to learn about different cultures (4)
	- Opportunity to think about the application of cultural nursing in clinical practice (3)
	Considering from the perspective of the client and gaining a new broader perspective (7)
	- Experience a new and broadening change in my perspective to understand clients (3)
	- Opportunity to think about nursing from the client’s point of view (2)
	- Reduction of prejudice against other cultures (2)
	Opportunities for learning and reflection by discussing various cultural nursing practices with colleagues (6)
	- Sharing and reflecting on cases of care for patients of different cultures with colleagues (5)
	- Opportunities for reflection on differences and discrimination (1)
	Strengthening cultural nursing competence (4)
	- Strengthening cultural empowerment (2)
	- Caring for people from other cultures has become somewhat more comfortable (1)
	- Greater tolerance for foreigners (1)
Comments on improvements	- Assignment burden (2)
	- Increasing the proportion of time spent sharing nursing case experiences with subjects with diverse cultures (1)
	- Further discussion of specific cultural nursing interventions that can be applied clinically (1)
	- Opportunities to meet interpreters and people from different cultures in person (1)
	- Audio issues in online lectures (2)

Participants’ comments were multiple responses.

## Footnotes

**Data availability**
Data files are available from Harvard Dataverse: https://doi.org/10.7910/DVN/U7BPLD Dataset 1. The response data from participants.

## Supplementary materials

Supplementary files are available from Harvard Dataverse: https://doi.org/10.7910/DVN/U7BPLD

Supplement 1. Survey questionnaire in Korean.

Supplement 2. Survey questionnaire translated into English.

Supplement 3. Content of the transcultural nursing course in the first semester in 2023 of the Graduate School of Nursing Education of Hallym University, Korea.

Supplement 4. Results of the paired t-test for 7 subscales of the questionnaire.

Supplement 5. Participants’ comments after completing the course in Korean.

Supplement 6. Audio recording of the abstract.
