# Supplementary material for: Effect of a transcultural nursing course on improving the cultural competency of nursing graduate students in Korea: a before-and-after study
Source: J Educ Eval Health Prof. 2023 Dec 4;20:35. doi: 10.3352/jeehp.2023.20.35 (PMC10955218; doi:10.3352/jeehp.2023.20.35)
Supplement: Supplementary file 2 — Supplement 1. Survey questionnaire in Korean. [file jeehp-20-35-suppl1.docx]

**Supplement 1.** Survey questionnaire in Korean

**Cultural Competence Scale for Registered Nurses**

**Demographic data**

학번:

귀하는 나이는? (만)___세

임상간호실무 경력은? (총___년)

지위:

근무병원:

근무부서:

근무하고 있는 부서는 다문화 대상자를 간호한 경험이 있습니까?

다문화 대상자를 간호한 경험이 있다면, 구체적으로?

문화 관련 교육을 받은 경험이 있습니까?

문화 관련 교육을 받은 경험이 있다면, 구체적으로?

외국에서 거주한 경험이 있다.

다문화간호 교과에서 학습하기를 기대하는 것은?

날짜:

**Cultural competency**

간호사의 문화역량에 대한 질문입니다. “(1) 전혀 그렇지 않다, (2) 그렇지 않다, (3) 보통이다, (4) 그렇다, (5) 매우 그렇다”로 귀하의 생각과 가장 가까운 곳에 √하여 주시기 바랍니다.

이 질문지에서 ‘대상자’는 한국인과 인종, 민족, 언어 등 문화적 배경이 다른 모든 간호 대상자를 의미합니다. 문화적 배경이 다른, 모든 간호 대상자를 떠올리며...

**Communication**

1. 나는 대상자에게 치료 및 간호 방법 등을 설명한 후 대상자가 이를 정확하게 시행하는지 확인한다.

2. 나는 대상자가 이해할 수 있게 설명한다.

3. 나는 대상자에게 친숙한 매체와 자료를 이용하여 정보를 제공한다.

4. 나는 대상자의 한국어 구사능력에 맞추어 소통한다.

5. 나는 대상자의 한국어 의사소통의 유창성 정도에 따라 그림, 몸짓 언어 등을 사용하여 소통한다.

6. 나는 필요할 때에는 통역사, 통역기, 지인을 동원하여 대상자와 의사소통한다.

**Biocultural ecology and family**

7. 나는 대상자의 성별과 연령에 따른, 가족 내에서의 대상자의 역할을 확인한다.

8. 나는 대상자 가족의 건강 관련 목표나 우선순위를 확인한다.

9. 나는 문화집단에 따라 생물학적 차이가 건강에 미치는 영향을 안다. (예: 지중해성 빈혈, 말라리아 등의 발병 차이)

10. 나는 문화집단에 따른 신체적 차이를 고려하여 신체를 검진한다. (예: 피부색, 모발, 정상 체질량지수 등)

11. 나는 대상자의 문화를 고려하여 신체를 사정한다.

12. 나는 문화집단에 따라 다르게 나타나는 질병의 취약성, 발생, 진행과정에 따라 간호 계획을 세운다.

13. 나는 문화집단에 따른 흡연, 알코올 섭취, 약물 오남용 행위의 위험도가 다르다는 것을 안다.

14. 나는 문화집단에 따른 대상자의 일상적인 신체활동 부족이나 과잉의 위험을 간호에 고려한다.

15. 나는 문화집단에 따라 다른 안전행위에 대한 정보를 제공한다. (예: 안전벨트 매는 방법, 보호장비 착용수칙, 안전운전, 성병 예방조치 등)

**Dietary life**

16. 나는 대상자의 문화에 따른 식생활의 차이를 인정한다.

17. 나는 대상자의 문화를 고려하여 식이를 제공한다.

18. 나는 대상자가 음식에 부여하는 의미, 의식과 금기사항을 안다.

**Death rituals**

19. 나는 문화에 따라 죽음의 의미가 다르다는 것을 안다.

20. 나는 문화에 따라 죽음의 의미가 다르다는 것을 간호에 활용한다.

21. 나는 문화에 따라 죽음에 대한 애도방식이 다르다는 것을 안다.

**Spirituality**

22. 나는 대상자의 종교적 행위를 사정한다.

23. 나는 대상자의 종교에 따른 삶의 의미를 사정한다.

24. 나는 대상자의 종교를 고려하며 간호한다.

**Equity**

25. 나는 대상자를 편견 없이 대한다.

26. 나는 대상자를 간호할 때 나의 위치와 역할을 인지한다.

27. 나는 대상자에게 평등한 간호를 제공하려고 노력한다.

28. 나는 대상자를 한 인간으로, 있는 그대로 받아들인다.

29. 나는 대상자 간호에 관심이 있다.

**Empowerment and intermediation**

30. 나는 대상자를 간호하기 위해 문화간호에 대한 기술을 습득한다.

31. 나는 문화집단의 건강, 질병, 간호와 관련된 지식을 쌓는다.

32 나는 다문화 간호 경험을 통해 대상자에게 접근하는 법을 터득한다.

33. 나는 근무지에서 운영되는 다문화 정책을 대상자와 연계한다.

34. 나는 대상자에게 건강 관련 복지 혜택에 관한 정보를 제공한다.

35. 나는 대상자와 건강 관련 지원체계 사이에 중재자 역할을 한다. (예: 병원 내 의료진, 사회사업가, 의료보험전담자 등/또는 병원 외 지역 보건소, 직장의 의무실 등)

**Satisfaction to curriculum [only after the intervention]**

다문화간호 교과 관련 좋았던 것은 다문화간호 교과 관련 개선될 부분은?

다문화 교과의 만족 정도는?
