# Supplementary material for: Effect of a transcultural nursing course on improving the cultural competency of nursing graduate students in Korea: a before-and-after study
Source: J Educ Eval Health Prof. 2023 Dec 4;20:35. doi: 10.3352/jeehp.2023.20.35 (PMC10955218; doi:10.3352/jeehp.2023.20.35)
Supplement: Supplementary file 3 — Supplement 2. Survey questionnaire translated into English. [file jeehp-20-35-suppl2.docx]

**Supplement 2.** Survey questionnaire translated into English

**Cultural Competence Scale for Registered Nurses (in English)**

**Demographic data**

Student number:

What is your age? (years) ___ years old

How many years of clinical nursing practice experience do you have (total ___ years)?

Status (job title):

Hospital name:

Department name:

Does your department have experience caring for multicultural populations? (yes or no)

→ If you have experience in caring for multicultural populations, specifically describe it.

Do you have experience with cultural training education? (yes or no)

→ If you have had any cultural training, specifically described it.

You have lived in a foreign country. (yes or no)

What do you expect to learn in a multicultural nursing course?

Date (year, month, day)

**Questionnaires on the cultural competency**

It is a question about nurses’ cultural competence. Please √ the place closest to your thoughts with “(1) strongly disagree, (2) disagree, (3) moderately, (4) agree, and (5) strongly agree.”

In this questionnaire, “client” refers to all care recipients from different cultural backgrounds, including race, ethnicity, and language. Think all care recipients from different cultural backgrounds.

**Communication**

1. I explain treatment and care methods to the subject and then verify that the subject performs them correctly.

2. I explain in a way that the client can understand.

3. I provide information using media and materials familiar to the audience.

4. I communicate according to the Korean language proficiency of the target client.

5. I communicate using pictures, body language, etc., depending on the level of fluency of the subject’s Korean communication.

6. I will use interpreters, translators, and friends to communicate with the person when necessary.

**Biocultural ecology and family**

7. I verify the subject’s role in the family based on gender and age.

8. I identify the client’s family’s health-related goals or priorities.

9. I know the health implications of biological differences across cultural groups (e.g., differences in the incidence of thalassemia, malaria, etc.).

10. I examine my body, considering physical differences based on cultural groups (e.g., skin color, hair, average body mass index, etc.).

11. I assess the body, considering the subject’s culture.

12. I plan care based on the vulnerability, occurrence, and course of diseases that differ among cultural groups.

13. I understand that different cultural groups are at other risk for smoking, alcohol consumption, and substance misuse behaviors.

14. I consider the risk of under- or over-activity of the client’s daily physical activity based on the cultural group in my nursing practice.

15. I provide information about safety behaviors that vary by cultural group (e.g., how to fasten seat belts, wear protective gear, safe driving, STD prevention measures, etc.).

**Dietary life**

16. I recognize differences in diet based on the subject’s culture.

17. I provide a diet that considers the client’s culture.

18. I know the meanings, rituals, and contraindications the subject attaches to food.

**Death rituals**

19. I recognize that death has different meanings in different cultures.

20. I utilize in my nursing practice that death has different meanings in different cultures.

21. I recognize that different cultures mourn death in different ways.

**Spirituality**

22. I assess the subject’s religious practices.

23. I assess the meaning of life according to the subject’s religion.

24. I provide care with consideration for the client’s religion.

**Equity**

25. I treat people in a nonjudgmental manner.

26. I recognize my position and role in the client’s care.

27. I provide equal care to my subjects.

28. I accept the person as a human being, as they are.

29. I am interested in subject care.

**Empowerment and intermediation**

30. I will acquire skills in cultural nursing to care for the client.

31. I learned about cultural groups’ health, illness, and care.

32 I learned to approach clients through multicultural nursing experiences.

33. I relate the multicultural policies of my workplace to my audience.

34. I provide the person with information about health-related benefits.

35. I act as an intermediary between the person and their health-related support system (e.g., medical staff, social workers, health insurance advocates, etc., in the hospital or community health centers outside the hospital, workplace health clinics, etc.).

**Satisfaction to curriculum [only after the intervention].**

What did you like about the multicultural nursing course that could be improved about the multicultural nursing course?

How satisfied are you with the multilingual classroom?
