# Supplementary material for: Effect of a transcultural nursing course on improving the cultural competency of nursing graduate students in Korea: a before-and-after study
Source: J Educ Eval Health Prof. 2023 Dec 4;20:35. doi: 10.3352/jeehp.2023.20.35 (PMC10955218; doi:10.3352/jeehp.2023.20.35)
Supplement: Supplementary file 4 — Supplement 3. Content of the transcultural nursing course in the first semester in 2023 of the Graduate School of Nursing Education of Hallym University, Korea. [file jeehp-20-35-suppl3.docx]

**Supplement 3.** Content of cultural nursing course in the first semester in 2023 of the Graduate School of Nursing Education of Hallym University, Korea

| Times | Domains | Specific themes and contents | Theoretical rationale | Methods |
| --- | --- | --- | --- | --- |
|  | Pretest | · Self-check of cultural competence scales for registered nurses |  | · Questionnaire |
| 1 | Introduction | · Orientation  · Organizing a small group and introducing members  · Understanding culture: definition and attributes of culture | Purnell’s model | · Lecture  · Group presentation |
| 2 | Culture and health | · Diversity and universality in transcultural nursing theory | Purnell’s model | · Lecture  · Group discussion |
| 3 |  | · Understanding health and nursing from a cultural perspective  · Various perspectives of health in different countries | Purnell’s model | · Lecture  · Group discussion |
| 4 | Human rights and respect for diversity | Cultural issues: difference, discrimination, and disparities  Discussion of news, articles, SNS, movies, books and issues caused by prejudice and stereotypes or cultural differences in our society | Purnell’s model | · Lecture  · Presentation & discussion  · Reflective activities |
| 5 | Cultural competence | · Cultural nursing competence: cultural awareness, knowledge, sensitivity, and skill | Purnell’s model | · Lecture_online (1)  · Individual report |
| 6–7 | Cultural diversity | · Globalization and cultural diversity  · Domestic multi-cultural society in Korea: marriage immigrants, migrant workers, north Korean defectors, foreign students, etc. | Purnell’s model | · Lecture  · Group discussion  · Group presentation |
| 8 | Cultural assessment | · Communication: verbal & nonverbal communication, medical interpreting  · Space: intimate/personal/social distance zone, spatial behavior | Giger & Haddad’s model | · Lecture_online (2)  · Individual report |
| 9 |  | · Social organization: family, death rituals, spirituality  · Time: past, present, future oriented time | Giger & Haddad’s model | · Lecture_online (3)  · Individual report |
| 10 |  | · Environmental control: internal locus, external locus of control, cultural health practices  · Biological variation: genetics, disease vulnerability | Giger & Haddad’s model | · Lecture_online (4)  · Individual report |
| 11 | Cultural intervention | · Sharing groups experiences with married immigrant women, foreign workers, and children of multi-cultural family | Giger & Haddad’s model | · Presentation & discussion |
| 12 |  | Problem-based learning 1: cases of married Vietnamese women who immigrated to Korea | Giger & Haddad’s model | · Case study  · Group discussion & presentation  · Reflective activities |
| 13 |  | · Problem-based learning 2: cases of Korean women who immigrated to Australia | Giger & Haddad’s model | · Case study  · Group discussion & presentation  · Reflective activities |
| 14 | Wrap-up | · Strategies for improving cultural competence in patient care  · Evaluation of the course, self-reflection | Purnell’s model | · Group discussion  · Group presentation  · Reflective activities |
| 15 |  | Term test |  |  |
|  | Posttest | · Self-check of cultural competence scales for registered nurses |  | · Questionnaire |
